# MED11/373: Intranet-/Internet Software for Preparation and Presentation of Lectures in Dermatology

**DOI:** 10.2196/jmir.1.suppl1.e54

**Published:** 1999-09-19

**Authors:** H Höhn, S Karl, H Hamm, J Albert

**Affiliations:** ^1^Universität Würzburg, WürzburgGermany

**Keywords:** Image Processing, Lectures, Dermatology, Internet, Intranet, Education

## Abstract

**Introduction:**

A software package consisting of a semantic network and co-operating Java applications was created for supporting lectures in dermatology or other visually oriented medical disciplines. This work is based on the SENTIMED-project (SEmantic Net Toolbox for Images in Medical EDucation) of the Department of Dermatology and the Chair for Computer Science II, University of Würzburg. SENTIMED is part of the Bavarian government initiative "Multimedia in Education".

**Methods:**

The first building block of the package is a digital image archive in SENTIMED which consists of over 4000 high resolution slides. Three sizes thereof (up to 1024x768 pixels) are stored on a CD-ROM together with the semantic network for navigation and a set of Java applications for image retrieval and manipulation. The highest resolution formats (3072x2048 pixels) are available from a server in the intranet. The automated installation on a stand-alone/intranet PC requires 30 MB of disk space. The CD-ROM contents is protected against unauthorized access and copying by either a local or a network-dongle, i.e. one in the local parallel port of the stand-alone PC or one in the intranet-server which the other PCs use via the clinic network.

**Results:**

The semantic network can be presented and explored by a standard WWW Browser. It consists of an XML database, program-generated HTML pages, CGI scripts and Java applets. Entry points to the network are several alphabetical and hierarchical lists of e.g. diagnoses or skin regions. Context-sensitive links to external sources like PubMed or RxList give a comfortable access to internet-resources for further information-retrieval. Within a separate Java application lecturers can search the archive for collections of images with specified criteria (diagnoses, ICD code, localisation, etc.). Images can be compared, copied into intermediate repositories and finally ordered for the presentation. Powerpoint slides can be imported as well as any other JPEG and GIF images. Regions of interest in the pictures are marked to supply high resolution zooms from the image archive. These regions are then extracted from the original on the server and transferred via Intranet. The presentation tool offers features like an index page for all slides (a tableau of thumbnail formats), zooming of predefined areas by loading parts in higher resolution or ad-hoc by calculated zooms of any rectangular area from the image. The lecture is presented with a high resolution multimedia projector.

**Discussion:**

On top of a digital image archive a set of Java routines efficiently supports lecturers in dermatology to prepare and present lectures or conference contributions. The semantic network layer guides the fast retrieval of images and related information in the context of an Intranet/Internet.

